# Patient reported outcomes and implant survivorship after Total knee arthroplasty with the persona knee implant system: two year follow up

**DOI:** 10.1186/s12891-019-2470-y

**Published:** 2019-03-04

**Authors:** N. M. C. Mathijssen, H. Verburg, N. J. London, M. Landsiedl, M. Dominkus

**Affiliations:** 1 Department of Orthopedics, Reinier de Graaf Hospital, Reinier de Graafweg 5, 2625AD, Delft, The Netherlands; 2 Department of Orthopaedics, Harrogate and District Foundation Trust, Park Rd, Harrogate, Lancaster, HG2 7SX UK; 3 Department of Orthopedics, Orthopaedic Hospital Speising, Speisinger Str. 109, 1130, Vienna, Austria; 4 Sigmund Freud Private University, Freudpl. 1, 1020 Vienna, Austria

**Keywords:** Total knee arthroplasty, Osteoarthritis, Patient reported outcomes

## Abstract

**Background:**

More personalized implant designs for total knee arthroplasty might optimize the clinical outcome after surgery. One of these personalized implant designs is the Persona knee implant system (Zimmer Biomet, Warsaw, Indiana, USA). The primary objective of this study was to determine patient reported outcomes and implant survivorship of the Persona Knee system used in primary total knee arthroplasty, up to two years after surgery.

**Methods:**

From November 2013 to July 2016 consecutive patients undergoing primary total knee arthroplasty were enrolled in a prospective observational cohort study at three centers. Preoperatively, at 6 weeks, 6 months, 1 and 2 years after surgery, patients completed the Knee Injury and Osteoarthritis Outcome Score (KOOS), the Oxford Knee Score (OKS), the Knee Society Score (KSS, 2011, modified version) and the EQ-5D. Adverse Events were captured, assessed for relationship to device, and recorded in the study database. Furthermore, physical functioning was assessed by the orthopedic surgeon.

Repeated measures analyses were performed on PROM scores. Kaplan Meier was used to calculate survivorship of the Persona Knee Implant System.

**Results:**

A total of 146 total knee arthroplasties were performed. 61% (89/146) of the patients were female and mean age was 64.7 (± 6.9) years. Two years after surgery, one patient had a revision of the polyethylene insert because of a periprosthetic joint infection. Therefore, the Kaplan-Meier survival estimate at 2 years was 0.99 (0.95–1.00 95% CI).

OKS increased from 22.1 (95% CI 20.9–23.3) to 41.8 (95% CI 40.6–43.1) two years after surgery. Furthermore, all other PROMs also increased from before surgery to 2 year postoperatively.

**Conclusion:**

The Persona Knee implant is safe and effective and the clinical results up to two years after surgery are promising. PROMs results are very good; pain, function and quality of life all improved greatly after TKA. Further studies are needed to determine the long term clinical performance of the Persona prosthesis.

**Trial registration:**

Clinicaltrials.gov (NCT02337244). Registered June 1st, 2015. Retrospectively registered.

## Background

Osteoarthritis (OA) of the knee is a disabling, painful joint disease. Total knee arthroplasty (TKA) has been performed successfully to treat moderate to severe osteoarthritis. In the last decades, the number of TKAs and the prevalence of OA have increased severely and are expected to increase further because of obesity, older age and TKAs performed in younger patients [1–3]. Although TKA has demonstrated effectiveness with substantive and sustained improvement in quality of life, clinical performance in patients one year after TKA remains lower than for healthy adults [4]. These functional deficits might be attributed to altered kinematics of the replaced joint [5].

More personalized implant designs might optimize the clinical outcome. They demonstrate an improved fit to patient anatomy [6]. Therefore, these implants might be better in addressing the requirements patients have nowadays, with a more active lifestyle.

One of these personalized implant designs is the Persona knee implant system (Zimmer Biomet, Warsaw, Indiana, USA). The Persona knee implant system has an anatomical shape of the tibial component of the prosthesis, which might result in a better fit and less overhang of the tibial tray. In addition, the large component sizes offering 1 mm thickness increment of the inlays might lead to improved knee stability post- surgery.

Therefore, the primary objective of this study was to determine patient reported outcomes and implant survivorship of the Persona Knee system used in primary total knee arthroplasty, up to two years after surgery. Secondary outcomes were complications and physical functioning.

## Methods

This prospective observational multicenter cohort study included patients who underwent primary cemented total knee arthroplasty. All patients were included consecutively between November 2013 and July 2016 at the participating hospitals (Reinier de Graaf Hospital, Delft, the Netherlands; Orthopadisches Spital Speising, Vienna, Austria; Harrogate and District NHS Foundation Trust, Harrogate, United Kingdom).

Inclusion criteria were patients aged between 18 and 75 years old, diagnosed with rheumatoid arthritis, osteoarthritis, traumatic arthritis, collagen disorders and/or avascular necrosis, post-traumatic loss of joint configuration, moderate valgus, varus or flexion deformities. Patients were excluded if currently participating in any other surgical intervention study or pain management study; a history of infection in the affected joint and/or other local/systemic infection that may affect the prosthetic joint; insufficient bone stock on femoral or tibial surfaces; skeletal immaturity; neuropathic arthropathy; osteoporosis or any loss of musculature or neuromuscular disease that compromises the affected limb; stable, painless arthrodesis in a satisfactory functional position; severe instability secondary to the absence of collateral ligament integrity; rheumatoid arthritis accompanied by an ulcer of the skin or a history of recurrent breakdown of the skin, sensitivity or allergy to one or more of the implant materials; pregnancy or a member of a protected population (e.g. prisoner, mentally incompetent); previously received partial or total knee arthroplasty for the ipsilateral knee.

The study was approved by each of the local ethics committees of the participating hospitals and the study was registered in Clinicaltrials.gov (NCT02337244). All patients signed informed consent.

All participating orthopedic surgeons received education on the Persona Knee Implant System. Furthermore, they all performed surgeries with the Persona Knee Implant System before the start of the study. All surgeons were familiar with the NexGen Implant System; the instruments used during surgery for the NexGen are comparable to the instrument of the Persona; therefore we did not expect a learning curve.

All patients were placed under general or spinal anesthesia. Patients received a Persona Knee Implant. Surgical approach was either medial parapatellar or midvastus, according to the surgeon’s preference. Each patient received physiotherapy therapy (exercise therapy), analgesia and thrombo-embolic prophylaxis according to the protocol of the hospital in which they were treated.

Preoperatively, social demographic data (age, sex), ASA-classification and Body Mass Index (BMI) were obtained. During surgery, data collection included the duration of the operation from incision until wound closure and complications. Preoperatively, at 6 weeks, 6 months, 1 and 2 years after surgery, patients completed the Knee Injury and Osteoarthritis Outcome Score (KOOS) [7], the Oxford Knee Score (OKS) [8], the Knee Society Score (KSS, version 2011 [9]) and the EQ5D [10]. At these moments, radiographs of the knee were made. Furthermore, physical functioning was assessed by the orthopedic surgeon by standardized functional tests and questionnaires. Complications were defined as minor, major and related or not to the device or procedure.

### Statistical analysis

Repeated measures analyses were performed on PROM Scores and ROM using SAS 9.4 with the Proc Mixed procedure to determine differences between the different follow up moments. Repeated measures analyses is a robust statistical procedure that allows for missing data, in this case, patients who have not had the device implanted long enough to make later visits such as their two and three-year visits. Repeated measures also allows for between-time comparisons and allows for the non-independence of within-subject PROM scores. Additionally, repeated measures are not required to have a joint distribution that is exactly normal [11], particularly in large data sets where the sample size is greater than *n* = 30.

Kaplan-Meier was used to calculate survivorship. Kaplan-Meier is the most widely used statistical procedure to calculate survivorship in medical devices. It is a non-parametric procedure, that is, it does not rely on any assumed distribution of failures and outputs an estimated survivorship percentage by time.

All statistical tests where *P* < 0.05 are considered statistically significant.

## Results

**Fig. 1:**
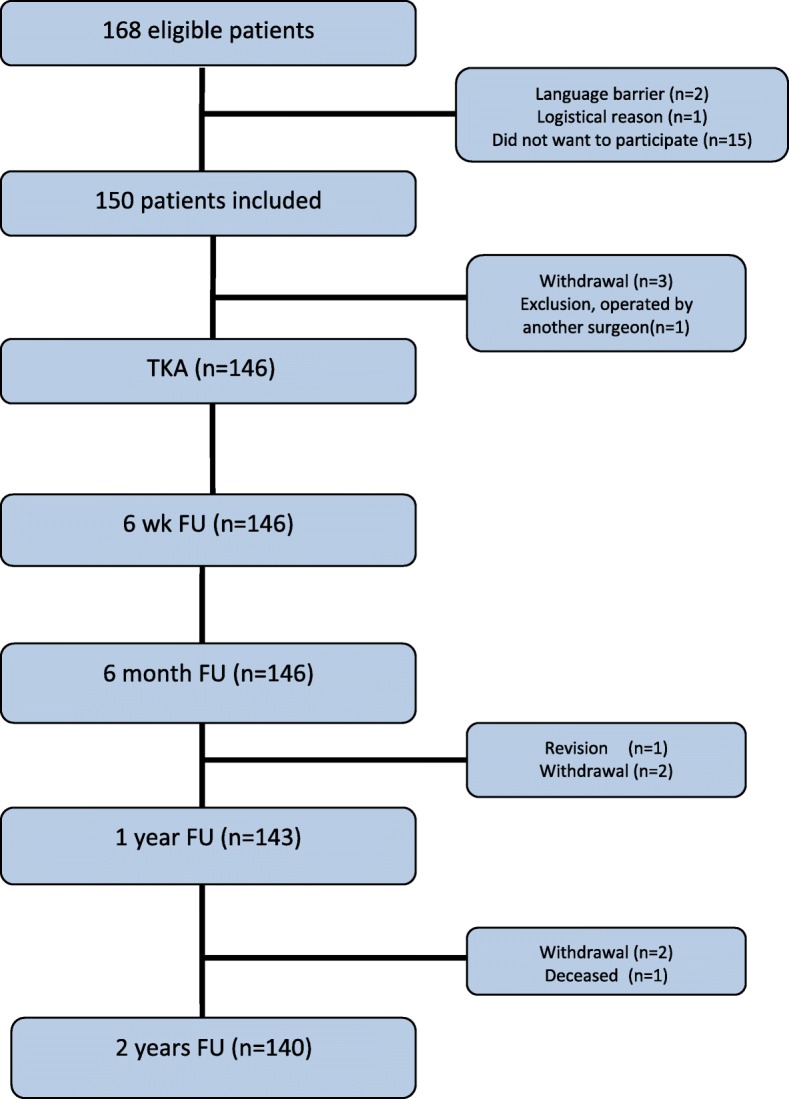
Inclusion of patients. Whiskers indicate the standard error

A total of 146 total knee arthroplasties were performed (Fig. 1). 123 patients (84.2%) received the Persona Posterior Stabilized knee implant (of which 23 patients received the Persona PS with narrow femoral implant configuration) and 23 patients (15.8%) received the Persona Cruciate Retaining knee implant 61% (89/146) of the patients were female and mean age was 64.6 (± 6.9). Patient characteristics and surgery details are shown in Table 1.

**Table 1:** Baseline patient characteristics and surgery details

Gender	Female	89 (61%)
	Male	57 (39.0%)
Age		64.6 (± 6.9)
BMI		30.3 (± 4.8)
Diagnosis	Osteoarthritis	142 (97.3%)
	Post-traumatic arthritis	4 (2.7%)
Operation Time (min)		74.3 (± 19.7)
Length of stay (days)		4.7 (± 3.1)
Surgical approach	Medial parapatellar	109 (74.7%)
	Midvastus	37 (25.3%)

Values are presented as mean (±SD), BMI = body mass index

### Survival

At two years follow up, 140 total knee arthroplasties were available for analysis (Fig. 1).

One patient had died because of cancer. One patient had a revision of the polyethylene insert because of a periprosthetic joint infection. Therefore, the Kaplan-Meier survival estimate for the prosthesis at 2 years was 0.99 (0.95–1.00 95% CI).

### Range of motion and PROMs

**Fig. 2:**
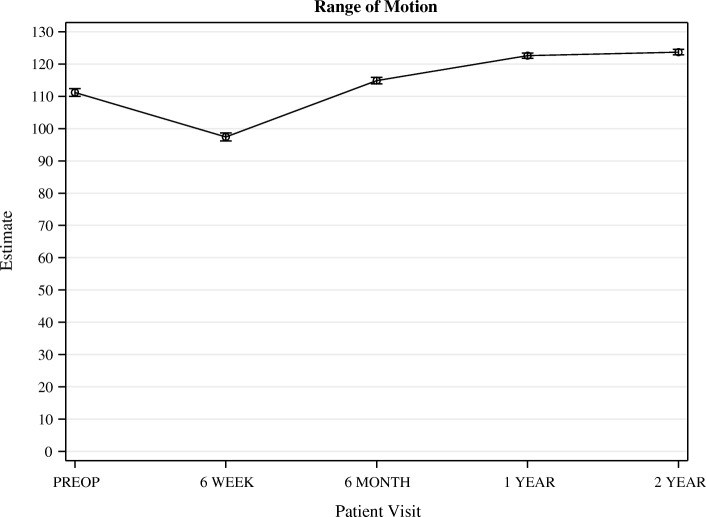
Range of Motion. Whiskers indicate the standard error

For the whole cohort, mean range of motion (ROM) was 111° (95% CI 109–113) before surgery. Six weeks after surgery, ROM had decreased significantly to 97° (95% CI 95–100). However, one year after surgery, ROM had increased significantly to 123° (95% CI 121–124, Fig. 2) and was stable up to two years follow up.

**Fig. 3:**
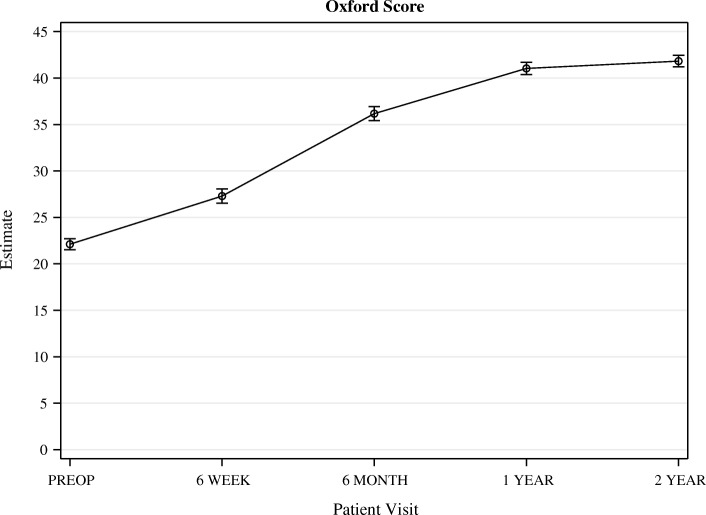
OKS. Whiskers indicate the standard error

**Fig. 4:**
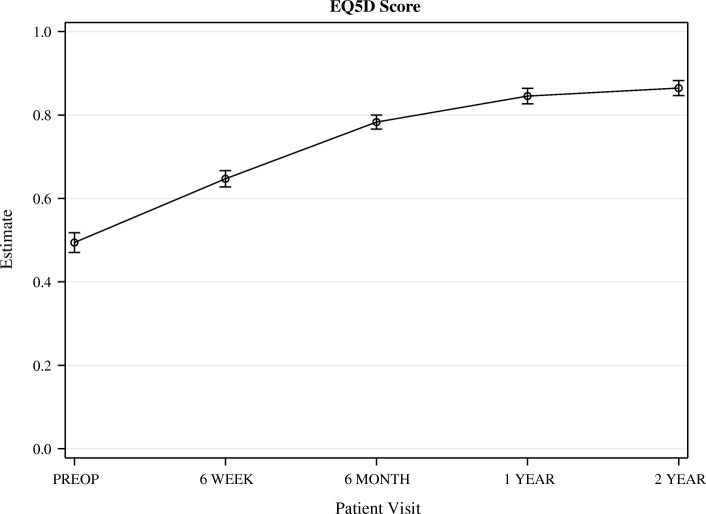
EQ5D. Whiskers indicate the standard error

**Fig. 5:**
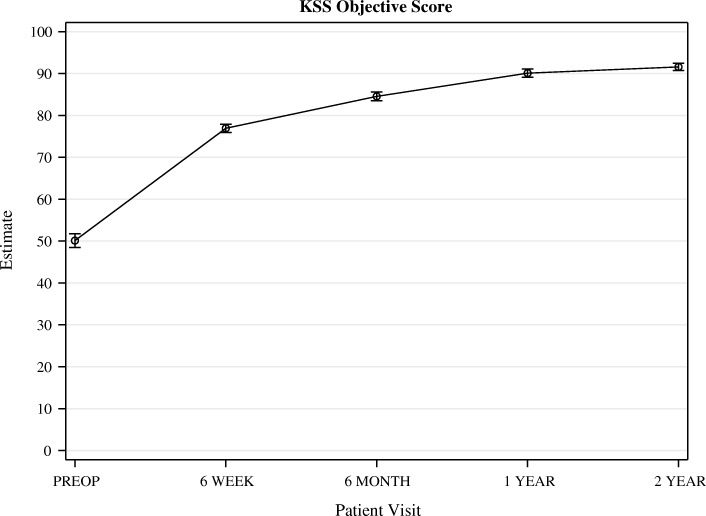
KSS Whiskers indicate the standard error

**Fig. 6:**
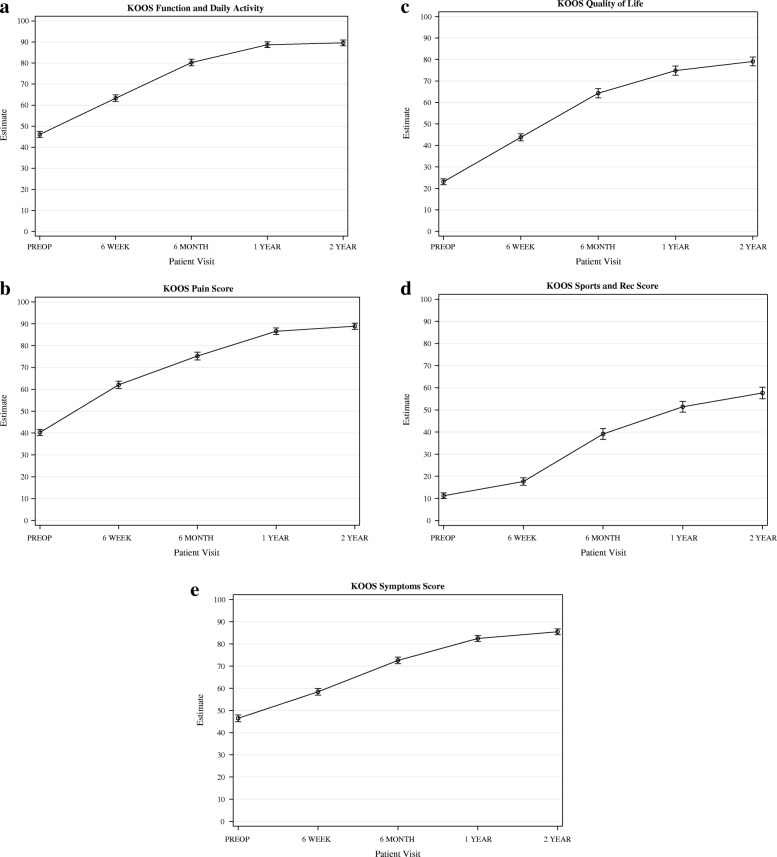
**a**.KOOS function and Daily Activity. Whiskers indicate the standard error. **b**. KOOS Pain. Whiskers indicate the standard error. **c**. KOOS Quality of Life. **d**. Whiskers indicate the standard error. KOOS Sports .Whiskers indicate the standard error. **e** KOOS symptoms.Whiskers indicate the standard error

All PROMs increased significantly from before surgery to 1 year postoperatively. From 1 to 2 years after surgery, no further significant increase could be observed (Figs. 3, 4, 5, 6).

OKS increased from 22.1 (95% CI 20.9–23.3) points before surgery, to 41.0 (95% CI 39.7–42.3) one year after surgery. Quality of life, measured with the KOOS as well as the EQ5D, had increased significantly 6 weeks after surgery and continued to increase until one year after surgery. KOOS pain was 40.3 (95% CI 37.5–43.0) points before surgery and increased significantly to 86.6 (95% CI83.7–89.5) at one year follow up.

### Complications

One patient had substantial disability 6 weeks after surgery. Therefore, the operated knee was manipulated under spinal anesthesia. Another patient had an injection with corticosteroids for persistent pain. A third patient had intra-articular drainage of the hematoma and wound irrigation for a thick painful knee with fever and increased CRP. All complications are shown in Tables 2 and 3.

**Table 2:** Major complications

Complication	Number of patients
Wound leakage	2 (1.4%)
Periprosthetic infection and insert revision	1 (0.7%)
Death	1 (0.7%)
Thick painful knee; intra-articular drainage of hematoma and wound irrigation	1 (0.7%)
Substantial disability 6 weeks after surgery; moving under spinal anesthesia	1 (0.7%)
Increased pain with injection of corticosteroids	1 (0.7%)

**Table 3:** Minor complications

Complication	Number of patients
Pain	12 (8.2%)
Urinary retention; catheter inserted	5 (3.4%)
Swelling of the knee	5 (3.4%)
Rash on knee after surgery	3 (2.1%)
Instability	1 (0.7%)
Urinary tract infection	1 (0.7%)
Fever after surgery; discharge delayed	1 (0.7%)
Fall with pain in knee	1 (0.7%)

## Discussion

The primary objective of this study was to determine patient reported outcomes and implant survivorship for the Persona knee implant system used in primary cemented total knee arthroplasty.

Within two years after surgery, one patient had a periprosthetic joint infection for which debridement was performed and the insert was exchanged, resulting in a survival estimate of 0.99. Although a survival estimate of 0.99 is good, we will need a longer follow up to draw definite conclusions on the performance of the Persona Knee System regarding the implant survival. Survival rates of total knee arthroplasties have improved in the last decades; the Swedish Knee arthroplasty Register showed an improvement in 10-year survival from 89% for TKAs performed during 1985 to 1994 to 96% during 2005 to 2014 [12]. Furthermore, from the Norwegian Arthroplasty Register it was concluded that the 10-year survival had improved to 94% in the period 2005–2015. [13] The explanation for these increasing survival rates is multifactorial, with adjusted patient selection, improved implant designs and altered education.

The number of complications in this study is low, which is in accordance with literature [14, 15]. Pain after total knee replacement is a known problem; between 7 and 20% of all operated patients have persistent pain after TKA [16].

Patient reported outcome measures were promising in this study; all measures improved significantly up to one year after surgery. The OKS, one year after surgery in this study (41.1 points) is considerably higher when compared to other TKA cohorts [17–19]. Moreover, the results of the OKS in the current study are comparable to the results of the OKS of a partial knee arthroplasty [20, 21].

An increase by more than the minimal clinically important difference (MCID) is more important than a significant difference since it reflects the clinical relevance of the increase. According to the study of Monticone et al., the MCID of KOOS for patients who underwent a TKA is 16.7 for Pain, 10.7 for Symptoms, 18.4 for ADL, 12.5 for Sports and 15.6 for Quality of Life. [22] However, Collins et al. conclude in a review that a change of at least 20 for all subscales represents a true change in older patients. [23] The results of the present study show that after six months, all subscales had an increase of at least 20 points. The MCID of the OKS is 5 points according to Clement et al. (2014) and 9 points according to Beard et al. (2015), therefore the increase of the OKS in the current study from before surgery to one year after surgery can be considered clinically relevant. [24, 25] No further increase between one and two year follow up could be observed. This is in accordance with the study of Matharu et al. (2014), who stated that one year after a TKA no relevant changes in OKS can be seen. [26] Nilsdottir et al. (2009) concluded that PROMs concerning pain and physical functioning are best after 12 months and that there is a decline in outcome from 1 to 5 years after TKA, although results are significantly better compared to before surgery. In the current study no decline in PROMs from one to two years after surgery was observed.

KSS also improved by more than the MCID, 6 and 12 months after surgery. The MCID of the KSS is 34.5 points [27] and the mean improvement of KSS was 34.5 and 40.1 points, respectively.

Judge et al. (2012) considered a 11-point change or more on OKS, six months after surgery, to be associated with high patient satisfaction. Furthermore, a score of 30 points or more on the OKS was related to the highest level of patient satisfaction six months after surgery [28].

In the current study, 126 patients (86%) had a 11-point change or more on the OKS or a score of 30 points or more, six months after surgery, indicating that the majority of patients was highly satisfied with the results of the surgery.

*Patient* related outcome measures become increasingly important in defining success of TKA [24]. According to Baker et al. [29], implant brand and hospital type were the only surgical factors influencing the improvement of PROMs after TKA, although the effect of these factors was small and not as pronounced as several patient factors.

Several limitations of this study need to be addressed. First, since this is a observational cohort study, comparison with other prosthesis is difficult. Second, although results of the current study are promising, the number of patients with two years follow up is relatively low as well as the years of follow up. Longer follow up and further studies are needed to determine long term survival and long term clinical performance of the Persona Knee implant.

## Conclusion

The Persona Knee implant is safe and effective and the clinical results up to two years after surgery are promising. PROMs results are very good; pain, function and quality of life all improved greatly after TKA. Further studies are needed to determine the long term results of the Persona prosthesis.

## Abbreviations

BMI: Body Mass Index
EQ-5D: EuroQuol-5D
KOOS: Knee Injury and Osteoarthritis Outcome Score
KSS: Knee Society Score
MCID: minimal clinically important difference
OA: Osteoarthritis
OKS: Oxford Knee Score
PROMs: Patient Reported Outcomes
ROM: range of motion
TKA: total knee arthroplasty
